# Lower regulatory frequency for postural control in patients with fibromyalgia and chronic fatigue syndrome

**DOI:** 10.1371/journal.pone.0195111

**Published:** 2018-04-04

**Authors:** Omid Rasouli, Ottar Vasseljen, Egil A. Fors, Håvard W. Lorås, Ann-Katrin Stensdotter

**Affiliations:** 1 Faculty of Medicine and Health Sciences, Department of Neuromedicine and Movement Science, Norwegian University of Science and Technology (NTNU), Trondheim, Norway; 2 Faculty of Medicine and Health Sciences, Department of Public Health and Nursing, Norwegian University of Science and Technology (NTNU), Trondheim, Norway; Public Library of Science, UNITED KINGDOM

## Abstract

As many similar symptoms are reported in fibromyalgia (FM) and chronic fatigue syndrome (CFS), underlying defcits may potentially also be similar. Postural disequilibrium reported in both conditions may thus be explained by similar deviations in postural control strategies. 75 females (25/group FM, CFS and control, age 19–49 years) performed 60 s of quiet standing on a force platform in each of three conditions: 1) firm surface with vision, 2) firm surface without vision and, 3) compliant surface with vision. Migration of center of pressure was decomposed into a slow and a fast component denoting postural sway and lateral forces controlling postural sway, analyzed in the time and frequency domains. Main effects of group for the antero-posterior (AP) and medio-lateral (ML) directions showed that patients displayed larger amplitudes (AP, *p* = 0.002; ML, *p* = 0.021) and lower frequencies (AP, *p* < 0.001; ML, *p* < 0.001) for the slow component, as well as for the fast component (amplitudes: AP, *p* = 0.010; ML, *p* = 0.001 and frequencies: AP, *p* = 0.001; ML, *p* = 0.029) compared to controls. Post hoc analyses showed no significant differences between patient groups. In conclusion, both the CFS- and the FM-group differed from the control group. Larger postural sway and insufficient control was found in patients compared to controls, with no significant differences between the two patient groups.

## Introduction

Similar symptoms reported in conditions defined by unexplained pain and fatigue such as fibromyalgia (FM) and chronic fatigue syndrome (CFS), albeit different diagnoses, may potentially occur due to similar underlying deficits. The intricate connectivity in the central nervous system (CNS) [1], suggests that many symptoms may be interconnected and pain and fatigue may directly or indirectly influence functional ability [2, 3], such as postural control [4–6]. Although FM is characterized foremost by pain and CFS mostly by fatigue, symptoms overlap with 50–70% and several diagnostic criteria are shared between conditions [7]. Pain and sensory amplification are common features and symptoms are characterized by great complexity in both diagnoses [8]. According to cognitive neuroscience, symptoms shared between these conditions may be an effect of chronification due to changes in similar pain regulatory mechanisms [9]. These changes may occur in unconscious domains, affecting both pain perception and motor control. Deficits in balance and postural steadiness have been demonstrated in patients with FM [10, 11] and disequilibrium has likewise been reported in individuals with CFS [12]. In a previous study, we reported similar deficits in dynamic postural control at gait initiation in both these patient groups [6].

In order to infer whether impairments in postural control may be explained by deficits in sensorimotor processing, systematic modulation of sensory systems allow the relative contribution of each system: visual, somatosensory and vestibular, to be examined [13]. To enable investigation on both the level of performance and on the level of control of posture, structural data analyses by decomposition of ground reaction forces registered by a force platform may be useful [14]. Center of pressure (CoP) measurements from the total reaction force can be decomposed into a slow, low-frequency component that represents the controlled parameter and a fast, high-frequency component that represents the controlling parameter [15]. The slow component is attributed to the motion of the body’s center of mass (CoM), i.e. postural sway, possibly reflecting supra-spinal postural control. The fast component represents the control mechanism for the location and motion of CoM and can be ascribed to the torque created by mechanical and reflex factors at the ankle joints [15, 16]. In addition to measures in the time domain that describe the magnitude of these components, additional measurements in the frequency domain are necessary to define the control strategies [17].

With evidence of reduced postural steadiness and disequilibrium in FM and CFS as a starting point, the aim of this study was to investigate similarities and dissimilarities measured on the performance level for postural sway and underlying control strategies between FM and CFS compared to controls. The rationale for the present study was to contribute to the knowledgebase about FM and CFS for a better understanding of symptoms that appear in the process of chronification of pain and fatigue.

## Materials and methods

### Participants

Seventy-five females, age 19–49 years, participated in this study (Table 1). The rationale for the choice of cohort was that the majority diagnosed with either CFS or FM are young to middle-aged women. Patients coming to the clinic were informed about the project and those interested were referred for participation by their attending physician and included consecutively over a period of 20 months. Diagnoses were determined in collaboration between a rheumatologist, psychiatrist and neurologist at the National Competence Center for Complex Symptom Disorders. Patients were diagnosed with either CFS according to the Center for Disease Control and Prevention criteria [18] or with FM according to the American College of Rheumatology (ACR) 1990 criteria [19]. Patients with comorbidity of CFS and FM were excluded. Eighty-seven patients (45 CFS and 42 FM) were found eligible for participation. Of those, 18 with CFS and 14 with FM declined. Due to technical issues compromising data quality, two data sets from each patient group were excluded. One patient with FM was not able to complete the tests due to pain. Data from 25 patients diagnosed with CFS and 25 patients diagnosed with FM was finally used for analyses. The severity of conditions was determined by the Fibromyalgia Impact Questionnaire (FIQ) [20] and the Chalder Fatigue Scale (CFS) [21] (Table 1). Twenty-five controls with no history of chronic pain or fatigue were recruited from students and staff of the hospital and university by announcement on the university and hospital intranet, and constituted an age- and gender-matched control group (CG). Exclusion criteria for both healthy participants and patients were diagnoses of psychiatric disorders, clinical depression, diagnosed neurological diseases including vestibular deficits, or musculoskeletal disorders (other than FM), or uncorrected reduced vision potentially interfering with postural control. Verbal and written information was given and written informed consent was obtained from each participant. The study was approved by the Regional Ethical Committee for Medical and Health Research Ethics (2012/679/REK midt) and conducted according to the Declaration of Helsinki.

**Table 1:** Characteristics of the participants in each group.

Variables	CG (N = 25)	CFS (N = 25)	FM (N = 25)
**Age (years)**	34.4 (7.9)	34.0 (8.9)	38.6 (8.0)
**Weight (kg)**	68.0 (9.8)	71.6 (12.9)	75.4 (14.3)
**Height (cm)**	167.2 (7.1)	169.1 (5.4)	168.5 (6.0)
**BMI (kg/m**^**2**^**)**	24.3 (3.5)	25.2 (5.1)	26.5 (4.5)
**Education (years)**	16.1 (2.3)	13.4 (2.5)	13.5 (2.2)
Pain level*	0.08 (0.28)	1 (1.16)	3.7 (1.8)
Fatigue level*	0.6 (0.8)	3 (1.8)	3.2 (2.2)
Chalder Fatigue Scale**	5.8 (5.7)	25.4 (3.8)	21.1 (5)
**FIQ**	-	-	56.9 (13)

Means (SD). CG: Control group, CFS: Chronic Fatigue Syndrome, FM: Fibromyalgia, FIQ: Fibromyalgia Impact Questionnaire.

*Level of pain and fatigue on the day of testing registered upon arrival to the lab using Numeric Rating Scale (0 no pain -10 worst pain).

** Level of fatigue using the continuous Chalder Fatigue Scale (0 no fatigue -33 most severe fatigue).

### Data acquisition

3D kinetic data was collected at 100 Hz with a force platform (Kistler Force Measurements, type 9260AA6, Kistler Instrument AG, Switzerland). Three different conditions of quiet standing were performed in the same and following order for all participants to ensure that potential effect of fatigue would be similar for all across trials: 1) firm surface with vision (VS), 2) firm surface without vision (NV), and 3) compliant surface (495 mm*406 mm*63 mm, Airex, AG, Switzerland) with vision (VS_c_). The duration of each separate test was 60 s and performed without shoes, feet parallel, and arms folded across the chest. Feet width was standardized as the distance equal to half the shoulder width measured between the acromial processes. Each participant performed all three conditions with the same foot position, marked on the platform before the first test. The participant was instructed to step onto the platform, and stand as still and relaxed as possible, and was further explicitly asked not to move the head or extremities, and maintain silence during testing. In the conditions with vision, a red cross (21 x 21 cm) placed 4 m away at the eye level served as a visual reference point. In the condition without vision, the participant was asked to keep the eyes closed. A blindfold was not advised due to the risk of sudden loss of balance. To establish a steady, quiet stance, the participant was informed that the test was commenced 10 s before the recording started and that it was finished 3 s after the data collection was completed. One-minute rest seated on a chair was provided between conditions.

### Data processing and analysis

Data from 58 s of each trial was used for further analysis. The first and the last second of the recording were excluded to avoid potential electronic noise from the start and stop key. All data (S1 Data) were analyzed in MATLAB (R2014a, MathWorks Inc., Natick MA). The signals were preprocessed with a Butterworth filter (8 Hz, low-pass, zero-lag, 2^nd^ order) and decomposed into a slow component for the controlled parameter of postural sway, and into a fast component for the controlling parameter attributed to ankle torque. Decomposition was calculated according to the concept of instant equilibrium forces as described by Zatsiorsky and Duarte [15, 16]. Instants of equilibrium points in the force signal when the total horizontal force equals zero were identified, and the CoP positions at these instants were determined and interpolated by a cubic spline function for estimation of the slow component. For estimation of the fast component, the deviation of CoP from the approximated curve of the slow component was determined.

The components were analyzed by computing the amplitudes and frequencies. The amplitude of each component was quantified by calculating the 95% confidence ellipse area (mm^2^) of CoP migration separately for each signal. The two axes of the ellipse were determined from the first two principal components from the analyses of the slow and fast components. The two radii of the ellipses were defined by the mediolateral (ML) and anteroposterior (AP) directions [22]. Mean power frequency spectra (Hz) for the ML and AP directions were estimated by a Fourier-analysis of the characteristics of the power spectral density using the Welch’s periodogram method [22].

### Statistical analysis

Data analyses were performed with SPSS statistical software (Version 22, IBM Corporation, USA). Normal distribution was verified with P-P plots, and histograms were used for control of skewness and kurtosis. One-way analysis of variance (ANOVA) was used to identify differences between groups in terms of characteristics. Repeated measures was used for main effects; with group as the between-subjects effect (n = 3; CG, CFS, and FM) and condition (n = 3; VS, NV and VS_c_) as the within-subjects factor. Corrections for sphericity using Greenhouse-Geisser were made when necessary. A mixed-design ANOVA was used to compare the effect of group on single variables in different conditions. Post-hoc pairwise comparisons with Bonferroni correction were employed to identify significant differences between pairs of groups, and pairwise comparisons between conditions within subjects. Partial eta-squared *η*^2^_*p*_ was used for effect size. The alpha level was established at p < 0.05.

## Results

No significant differences were found between the groups in terms of age, weight, height and BMI (Table 1). There were however significant differences between groups for the level of pain and fatigue on arrival to the lab, Chalder Fatigue scale and education, where pain was highest in FM and Chalder score was highest in CFS. Fatigue on arrival to lab was higher in patients, and patients had less years of education compared to controls (Tables 1 and 2).

**Table 2:** Post-hoc comparisons for the significant factors.

Variable	Group	Post-hoc	Mean difference
**Pain level on arrival to lab**	F _(2,72)_ = 59.2	CG-CFS	-0.9*
		CG-FM	-3.6**
		CFS-FM	-2.7**
**Fatigue Level on arrival to lab**	F _(2,72)_ = 18.9	CG-CFS	-2.4**
		CG-FM	-2.6**
**Chalder Fatigue Scale**	F _(2,72)_ = 108.9	CG-CFS	-19.5**
		CG-FM	-15.3**
		CFS-FM	4.2*
**Education, years**	F _(2,72)_ = 10.7	CG-CFS	2.7**
		CG-FM	2.5**

CFS: Chronic Fatigue Syndrome, FM: Fibromyalgia, CG: Control group.

* = *p*<0.05,

** = *p*<0.001

In general, patients with CFS and FM showed consistently larger amplitudes and lower frequencies compared to CG for both the slow and fast components across all three conditions. Table 2 lists the results of interactions, main effects and post hoc comparisons for all variables. An interaction between group and condition for the amplitude of the fast component in ML *(p* < 0.001) showed a different pattern for FM and CFS compared to CG, where the amplitude of the fast component increased more from VS to NV in the patient groups compared to in the control group. No statistical differences were found between CFS and FM on any variable in any condition.

### The slow component: Postural sway

**Fig 1:**
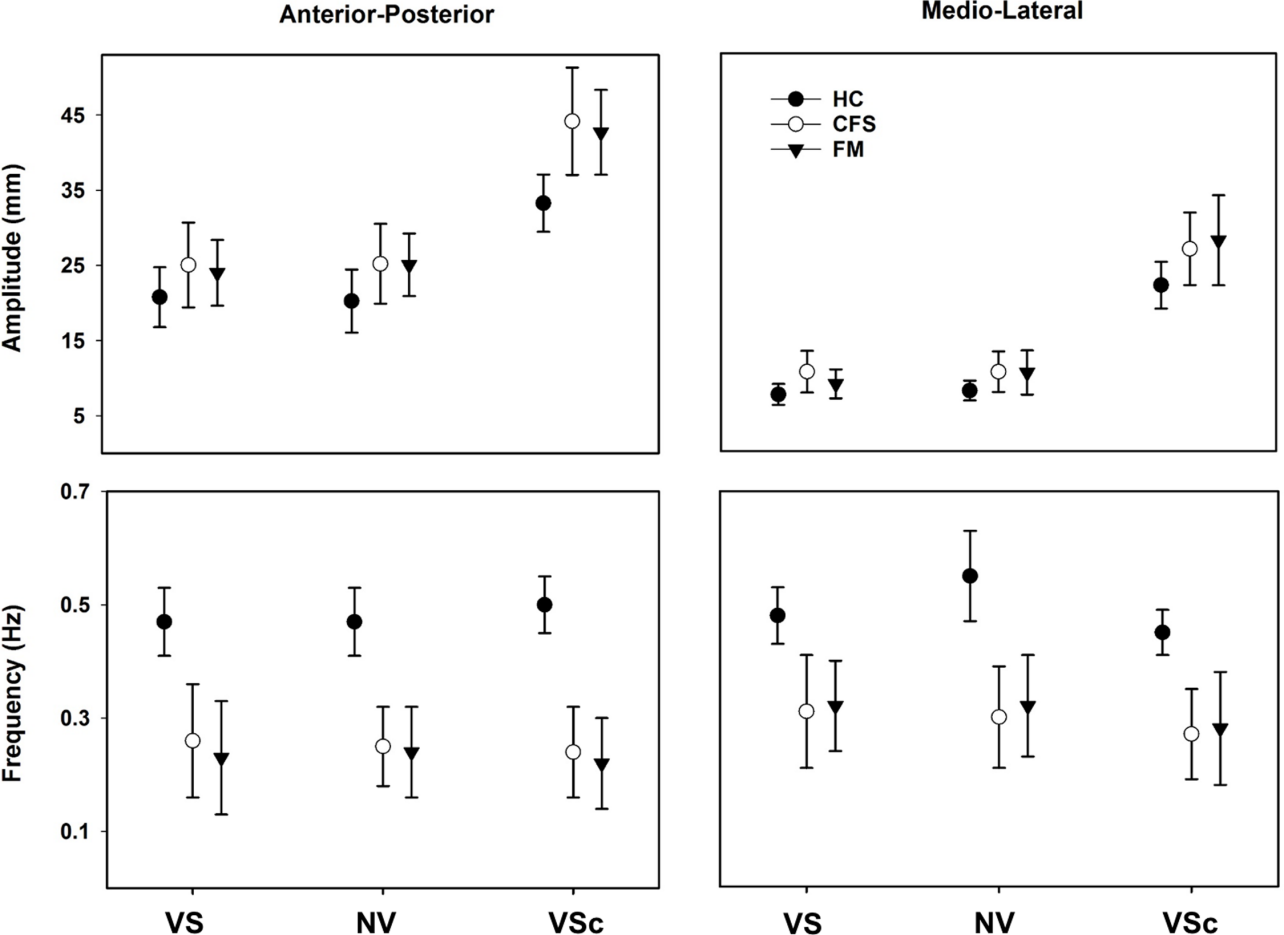
Estimated group means and SD for the slow component for each condition and both antero-posterior and medio-lateral directions during quiet standing on firm surface with vision (VS), on firm surface with no vision (NV), and on compliant surface with vision (VS_c_) for Control group (CG), Chronic Fatigue Syndrome (CFS), and for Fibromyalgia (FM).

There was a significant main effect of group for amplitude (AP, *p* = 0.002; and ML, *p* = 0.021) and frequency (AP, *p* < 0.001; and ML, *p* < 0.001) in both directions. Fig 1 shows that the amplitudes were similar in both patient groups but larger than in CG, in particular in the AP direction. Likewise, the frequencies were similar in both patient groups but lower than in CG. Post hoc analysis showed significant differences between FM-CG and between CFS-CG (Table 3), revealing larger amplitudes and lower frequencies in both patient groups compared to CG (Fig 1, Table 3). Within-subjects comparisons revealed a significant main effect of condition for amplitude (AP, *p* < 0.001; and ML, *p* < 0.001) in both directions and of frequency (ML, *p* = 0.001) in one direction. Pairwise comparisons between conditions showed a significant increase of amplitude for VS_c_ compared to VS and NV in both directions (*p* = < 0.001 for all), and a significant decrease of frequency for VS_c_ compared to VS and NV (*p* = 0.012, *p* = 0.002, respectively) in ML. There was no statistical difference in amplitude or frequency between the first two conditions: VS and NV. Overall, the VS_c_ condition showed the largest amplitude and lowest frequency for all groups.

**Table 3:** Interactions, main effects of condition and group with post hoc comparisons for migration of center of pressure for the slow and fast components during three conditions of quiet standing on a force platform on a firm surface with and without vision, and on a compliant surface with vision.

	Variable	Group*Condition	Condition	Group	Post hoc
**Slow component**	**Amp. AP**	F_(3.5,124.5)_ = 1.27 *η*^*2*^_*p*_ = 0.034	**F**_**(1.7,124.5)**_ **= 88.43** ***η***^**2**^_***p***_ **= 0.551**	**F**_**(2,72)**_ **= 6.82** ***η***^**2**^_***p***_ **= 0.159**	**CG-CFS** **CG-FM**
	**Amp. ML**	F_(2.9,104.1)_ = 1.83 *η*^2^_*p*_ = 0.032	**F**_**(1.4,104.1)**_ **= 207.60** ***η***^**2**^_***p***_ **= 0.742**	**F**_**(2,72)**_ **= 4.91** ***η***^**2**^_***p***_ **= 0.12**	**CG-CFS**
	**F. AP**	F_(4,144)_ = 0.961 *η*^2^_*p*_ = 0.026	F_(2,144)_ = 0.005 *η*^2^_*p*_ = 0.001	**F**_**(2,72)**_ **= 25.22** ***η***^**2**^_***p***_ **= 0.41**	**CG-CFS** **CG-FM**
	**F. ML**	F_(4,144)_ = 1.69 *η*^2^_*p*_ = 0.045	**F**_**(2,144)**_ **= 8.07** ***η***^**2**^_***p***_ **= 0.101**	**F**_**(2,72)**_ **= 14.74** ***η***^**2**^_***p***_ **= 0.29**	**CG-CFS** **CG-FM**
**Fast component**	**Amp. AP**	F_(2.9,104.4)_ = 0.47 *η*^2^_*p*_ = 0.013	**F**_**(1.4,104.4)**_ **= 99.02** ***η***^**2**^_***p***_ **= 0.579**	**F**_**(2,72)**_ **= 4.9** ***η***^**2**^_***p***_ **= 0.12**	**CG-CFS** **CG-FM**
	**Amp. ML**	**F**_**(2.7,96.8)**_ **= 6.29** ***η***^**2**^_***p***_ **= 0.149**	**F**_**(1.3,96.8)**_ **= 142.50** ***η***^**2**^_***p***_ **= 0.664**	**F**_**(2,72)**_ **= 7.91** ***η***_***p***_^**2**^ **= 0.18**	**CG-CFS** **CG-FM**
	**F. AP**	F_(4,144)_ = 0.82 *η*^2^_*p*_ = 0.022	F_(2,144)_ = 2.95 *η*^2^_*p*_ = 0.039	**F**_**(2,72)**_ **= 8.72** ***η***^**2**^_***p***_ **= 0.195**	**CG-CFS** **CG-FM**
	**F. ML**	F_(4,144)_ = 0.78 *η*^2^_*p*_ = 0.021	F_(2,144)_ = 2.86 *η*^2^_*p*_ = 0.038	**F**_**(2,72)**_ **= 3.73** ***η***^**2**^_***p***_ **= 0.09**	**CG-FM**

Amp: Amplitude (mm), F: Frequency (Hz), AP: Antero-posterior, ML: Medio-lateral. CFS: Chronic Fatigue Syndrome, FM: Fibromyalgia, CG: Control group. Bold text = significant. Post hoc: only significant comparisons are listed. Effect size: *η*^2^_*p*_ (small = 0.01; medium = 0.06; large = 0.14).

### The fast component: Control of postural sway

**Fig 2:**
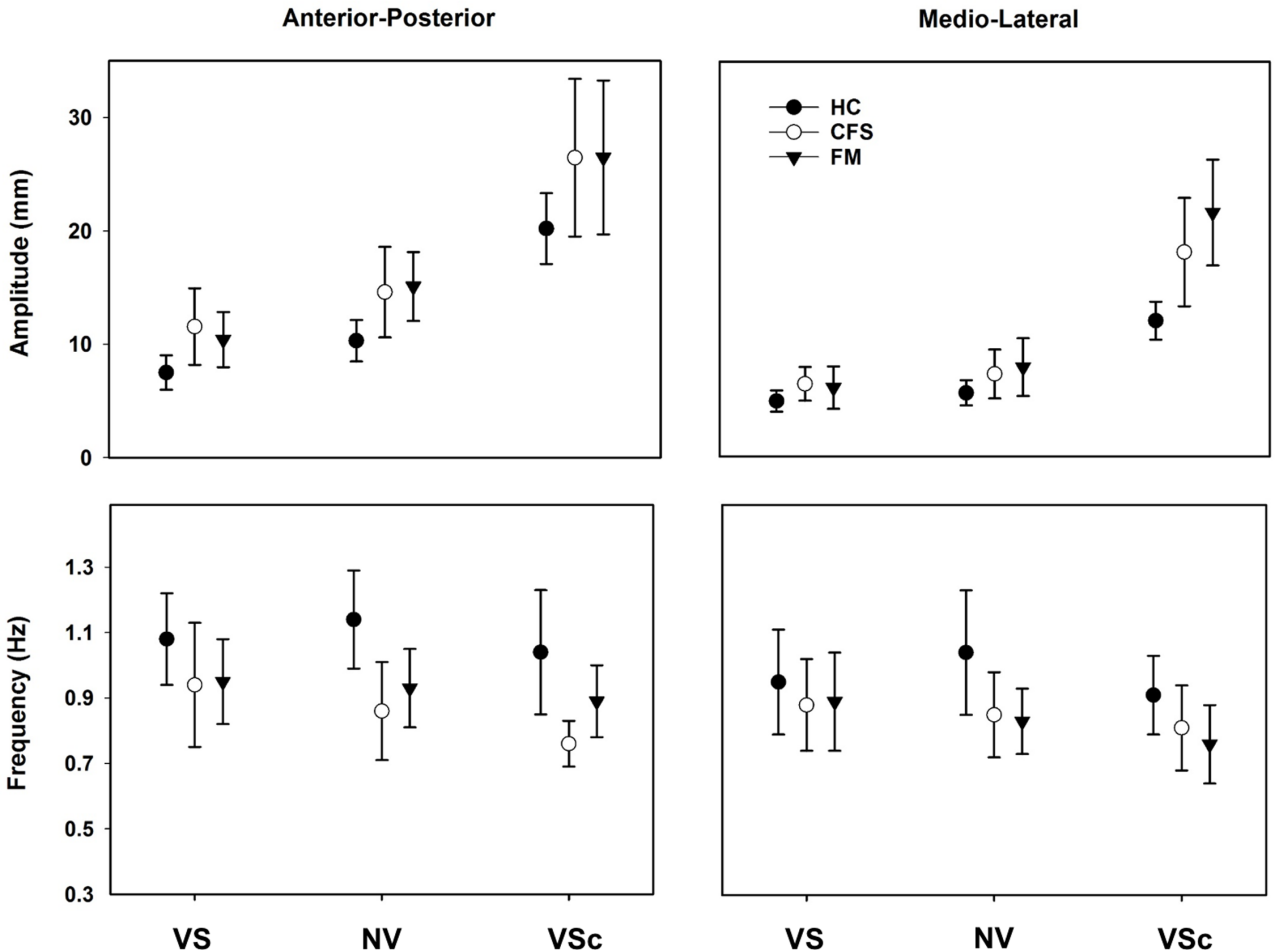
Estimated group means and SD for the fast component for each condition and both antero-posterior and medio-lateral directions during quiet standing on firm surface with vision (VS), on firm surface with no vision (NV), and on compliant surface with vision (VS_c_) for Control group (CG), Chronic Fatigue Syndrome (CFS), and for Fibromyalgia (FM).

A significant main effect of group was found for amplitude (AP, *p* = 0.010; and ML, *p* = 0.001) and frequency (AP, *p* < 0.001; and ML, *p* = 0.029) in both directions. Fig 2 shows similar amplitudes in both patient groups that were larger compared to CG, and similar frequencies in both patient groups but lower than in CG. Post-hoc analyses showed significantly larger amplitudes, and lower frequencies for FM and CFS compared to CG (Table 2). Within subjects’ comparisons, showed a significant main effect of condition only for amplitude (AP, *p* < 0.001; and ML, *p* < 0.001). Pairwise comparisons between conditions showed that the amplitude was increasing significantly from VS to NV and from NV to VS_c_ in the AP direction (*p* < 0.001 for both). In the ML direction the amplitude increased from VS to NV (*p* = 0.023) and from NV to VS_c_ (*p* < 0.001) (Fig 2, Table 3).

## Discussion

The present study is the first to collectively assess postural control in quiet standing with modulation of sensory information in patients with FM and CFS compared to healthy individuals, including both the controlled variable, i.e., postural sway (slow component) and the variable that controls postural sway (fast component). We observed a similar and consistent pattern of larger amplitudes and lower frequencies for both the controlled and the controlling variables in both patient groups in all three conditions (VS, NV and VS_c_). This suggests that patients used a different strategy compared to CG to control posture which was less efficient and resulted in greater postural sway. An interaction between condition and group showed a greater increase in the amplitude of the fast component in patients when vision was removed, demonstrating that patients used greater increase of ankle torque to control the location and movement of CoM, i.e., postural sway, in the absence of visual information, indicating a deficit in somatosensory information processing. This was further supported by the greatest group difference demonstrated for VS_c_ that showed that maintaining postural steadiness was more challenging in patients than CG when somatosensory information was modulated. Standing on a compliant surface attenuates information from proprioception and skin receptors [23], and visual information did not seem to compensate for the indicated deficits in somatosensory information. Although the outcomes in FM and CFS relative to CG varied between the patient groups, no significant differences were found between FM and CFS. These findings support our hypothesis that patients with FM and CFS would show different results from CG, but would display similar patterns of postural control to each other. Thus, this adds another feature to the list of similarities between FM and CFS [24].

The findings in the present study are also in agreement with previous studies on each patient group separately, showing impaired postural control in FM and CFS [10–12, 25]. Furthermore, Vaillant et al. (2016b) also found that the difference between FM and healthy subjects increased in the absence of visual information. Although mentioned studies have reported disequilibrium, poor balance and frequent falls in patients with FM or CFS, outcomes were on a performance level, and the underlying mechanisms were not investigated in these previous studies.

In the present study, structural analyses of CoP revealed in addition to outcomes on a performance level, also information about the strategies or mechanisms that control posture. This was achieved by decomposition of the CoP signal from quiet standing, were the fast component reflects the torque created by the movement of the ankle joint that generates the lateral forces, which control the location and movement of CoM [15]. From the present findings, it could be deduced from the fast component that patients produced a regulatory frequency which was too low and an excessive magnitude of ankle torque, possibly as a compensatory strategy, suggesting a general inverse relationship between amplitude and frequency. In theory, this strategy caused greater postural sway by pushing CoM too far in one direction before a counteracting force was produced to reverse the direction of the movement of CoM to keep the center of gravity within the limits of the base of support. Although unperturbed stance is based on a one-segment inverted pendulum model, and that the ankle joint movement gives a good estimate of the displacement of the center of mass, the true movement is multi-segmental [26]. The analysis of the fast and slow components takes this into consideration as the fast component of the CoP-signal is only in theory ascribed to the ankle joint. In reality, the fast component reflects any lateral force, regardless of source. Thus, the lower frequency and greater amplitude found in patients may also depend on a mechanical effect of movement distribution and synchrony across body segments.

Lower regulatory frequency has also been found for the control of upper limb position in FM [27]. Similar to postural control in the present study, upper limb control deteriorated more in the patients with FM than in healthy subjects when visual information was removed [28]. In general, these findings corroborate the results of the present study suggesting deficits in somatosensory information or sensorimotor processing and a general slowness in the motor control system.

Larger postural sway, revealed by larger amplitudes in the slow component, in patients may also be explained by the drift-and-act hypothesis, which also fits the findings of a general slowness demonstrated in lower regulatory frequency. This hypothesis suggests that postural control consists of sequences of drift-and-act episodes where the alignment of the body is deviating from the vertical line until sensory signals are processed in the CNS, and corrective actions are initiated [29]. Accordingly, CNS processing speed and time delay to trigger a corrective action is crucial. Longer processing time and slower action process may explain lower regulatory frequency and larger drift of CoM, i.e., postural sway, in both FM and CFS compared to CG. Recently, we published a study on dynamic postural control in a random sample of the present cohort. Those findings showed similar deficits in both patient groups in dynamic postural control during gait initiation [6]. Patients displayed a mismatch between position and velocity of CoP resulting in a short and abrupt deceleration phase toward the end of the first step, pushing CoM toward the limits of the base of support. This study supports the present results, and indicate a general deficit in static as well as dynamic postural control in both FM and CFS. These patterns suggest that there may be an underlying common denominator of deficits in sensorimotor control of perhaps similar origin in both patient groups.

Findings of similar deficits in FM and CFS on a performance level as well as on the level of control strategies does however not automatically mean that these conditions are one and the same. They may still be expressions of different pathologies. For example, in an earlier study by our group on psychotic patients exposed to a similar protocol, similar impairments were found on a performance level as well as in control strategies, characterized by increased amplitude of the slow and fast component, and lower regulatory frequency of the fast component. Likewise, the greatest difference compared to controls was found in quiet standing on a compliant surface [17]. In that paper, we proposed that the difficulties to estimate and correct the location and movement of CoM were dependent on deficits in sensory integrative and sensorimotor functions. This presumption was motivated by evidence of perturbed integrative function in the central nervous system in psychotic conditions [30]. Thus, similar deficits in postural control may occur in widely different diagnoses.

Pain intensity may influence sensorimotor dysfunction across different pain conditions as chronification appears to cause changes in similar regulatory mechanisms [9]. Higher perceived pain intensity is suggested to relate to lower level of postural automaticity, postural adaptability deficits to environmental challenge [31], and increased postural instability (i.e. increased postural sway) [32]. A vicious circle of pain and motor control deterioration may be generated [31] in line with the pain adaptation model [33].

Deficits in postural control in FM and CFS may be explained by perturbed sensorimotor processing. Patients with FM have shown reduced regional connectivity within the primary [34] and between the primary and secondary somatosensory cortex, with abnormal connectivity in visual cortices and reduced functional connectivity between the visual and the primary somatosensory cortex [35]. Reductions in task-evoked brain activation in visual areas, suggests attenuated responses early in sensory cortices [36]. Altered functional connectivity is also reported in CFS for several brain regions such as the cingulate cortex, and for connectivity between the sensory-motor and salient networks [37]. Thus, deficits in somatosensory information processing and possibly insufficient compensation by visual information may explain the present findings of a general slowness in the control strategy.

The slowness of response is also supported by evidence of longer reaction and movement times in both FM and CFS [2]. Further support for a neurophysiological basis of motor control deficits in FM and CFS have been demonstrated in behavioral tasks with transcranial magnetic stimulation revealing reduced corticospinal excitability [38], and indications of altered responsiveness of motor cortex and basal ganglia as well as dopaminergic irregularities [39–42]. Notably, with reference once again to similar results in our previous study on postural control in psychotic patients [17], dopaminergic dysregulation, which is typical in psychotic conditions, may perturb coordination between distributed neural networks [30]. Furthermore, studies on FM and CFS suggest that these changes may be attributed to the significant acceleration of age-related decrease in both white and gray matter [43, 44]. Interestingly, similar control strategies have been demonstrated during quiet standing in elderly who displayed greater magnitude of the fast component compared to young individuals. Furthermore, similar to the present results, the amplitude of the fast component increased relatively more in the elderly compared to the young individuals when vision was removed [45]. Although sites of gray and white matter reduction appear to differ between FM and CFS, the motor strategies during quiet standing bear similarities. Future studies should focus on a neurophysiological basis of direct evidence for a sensorimotor explanation for impaired postural control in these conditions, and whether that differs between FM and CFS.

Some limitations of the present study should be considered for interpretation and implication of the findings. The relatively small sample size may limit external validity due to the great heterogeneity of symptoms in both CFS and FM. Use of medication was not monitored beyond the general use of analgesics. Analgesics may potentially have affected performance positively because of reduced pain or negatively by possible side effects. Anti-depressive drugs were not prescribed to these patients.

In conclusion, differences in performance and postural control during quiet standing in patients with chronic fatigue syndrome and fibromyalgia varied relative to healthy controls, but with no significant differences between the patient groups. In general, patients displayed a different pattern of postural control. Differences in the fast and slow components was interpreted as lower regulatory frequency and greater ankle joint torque resulting in greater postural sway in patients. Both patient groups increased the ankle torque more than controls when vision was removed, and the largest difference between healthy individuals and patients occurred when somatosensory information was modulated with vision enabled. This suggests deficits in somatosensory information processing and possibly insufficient compensation from visual information. Findings of similar deficits in FM and CFS does however not automatically transfer to a similar origin of deficits.

## Supporting information

S1 Data Processed data for quiet standing on amplitude (AMP) and frequency (MF) for the slow (RM) and fast (TR) components in the anteroposterior (AP) and mediolateral (ML) directions for the three conditions: with vision (QO), without vision (QCL), and with vision standing on a compliant surface (QP) for the three groups: control (0), chronic fatigue syndrome (1), and fibromyalgia (2). (MAT)
